# 

**Published:** 1858-05

**Authors:** 


					﻿No. XII ]
MAY.
[Vol. XIII.
BUFFALO
MEDICAL JOURNAL
w
AND
|lbntl)b) Mmeiv*
CiF
• MEDICAL AND SURGICAL SCIENCE.
SANFORD B. HUNT. M. D.,
EDITC IR,
AUSTIN FLINT, Jr., M. D.,
ASSOCIATE EDITOR.
jf
BUFFALO, N. Y.:
E. R. J E IV E T T <fc CO.’S STEAM PRESS.
Commercial Advertiser Buildings.
1858.
Price $2.00 in advance, or $2.50 at the end of three montht
				

